# Predictors of persistent postoperative opioid use following colectomy: a population‐based cohort study from England

**DOI:** 10.1111/anae.16055

**Published:** 2023-06-02

**Authors:** R. M. Baamer, D. J. Humes, L. S. Toh, R. D. Knaggs, D. N. Lobo

**Affiliations:** ^1^ Division of Pharmacy Practice and Policy, School of Pharmacy University of Nottingham Nottingham UK; ^2^ Department of Pharmacy Practice, Faculty of Pharmacy King Abdulaziz University Jeddah Saudi Arabia; ^3^ Nottingham Digestive Diseases Centre and National Institute for Health Research Nottingham Biomedical Research Centre Nottingham University Hospitals NHS Trust and University of Nottingham, Queen's Medical Centre Nottingham UK; ^4^ Division of Pharmacy Practice and Policy, School of Pharmacy University of Nottingham Nottingham UK; ^5^ Division of Pharmacy Practice and Policy, School of Pharmacy University of Nottingham Nottingham UK; ^6^ Pain Centre Versus Arthritis University of Nottingham Nottingham UK; ^7^ Nottingham Digestive Diseases Centre and National Institute for Health Research Nottingham Biomedical Research Centre Nottingham University Hospitals NHS Trust and University of Nottingham, Queen's Medical Centre Nottingham UK; ^8^ David Greenfield Metabolic Physiology Unit, MRC Versus Arthritis Centre for Musculoskeletal Ageing Research School of Life Sciences University of Nottingham, Queen's Medical Centre Nottingham UK

**Keywords:** colectomy, England, epidemiology, opioids, persistent postoperative opioid use, risk factors

## Abstract

This retrospective cohort study on adults undergoing colectomy from 2010 to 2019 used linked primary (Clinical Practice Research Datalink), and secondary (Hospital Episode Statistics) care data to determine the prevalence of persistent postoperative opioid use following colectomy, stratified by pre‐admission opioid exposure, and identify associated predictors. Based on pre‐admission opioid exposure, patients were categorised as opioid‐naïve, currently exposed (opioid prescription 0–6 months before admission) and previously exposed (opioid prescription within 7–12 months before admission). Persistent postoperative opioid use was defined as requiring an opioid prescription within 90 days of discharge, along with one or more opioid prescriptions 91–180 days after hospital discharge. Multivariable logistic regression analyses were conducted to obtain odds ratios for predictors of persistent postoperative opioid use. Among the 93,262 patients, 15,081 (16.2%) were issued at least one opioid prescription within 90 days of discharge. Of these, 6791 (45.0%) were opioid‐naïve, 7528 (49.9%) were currently exposed and 762 (5.0%) were previously exposed. From the whole cohort, 7540 (8.1%) developed persistent postoperative opioid use. Patients with pre‐operative opioid exposure had the highest persistent use: 5317 (40.4%) from the currently exposed group; 305 (9.8%) from the previously exposed group; and 1918 (2.5%) from the opioid‐naïve group. The odds of developing persistent opioid use were higher among individuals who used long‐acting opioid formulations in the 180 days before colectomy than those who used short‐acting formulations (odds ratio 3.41 (95%CI 3.07–3.77)). Predictors of persistent opioid use included: previous opioid exposure; high deprivation index; multiple comorbidities; use of long‐acting opioids; white race; and open surgery. Minimally invasive surgical approaches were associated with lower odds of persistent opioid use and may represent a modifiable risk factor.

## Introduction

Colectomy is a common abdominal surgical procedure, with 300,000 performed annually in the USA [1] and approximately 33,000 performed annually in England [2]. People undergoing colectomy might have diseases that may be associated with pain, such as inflammatory bowel disease, diverticulitis and cancer [3]. Additionally, the procedure itself can lead to significant postoperative pain [3] and opioid analgesia may be indicated.

While short‐term opioid use has an established role in managing acute pain [4], it has recently been identified as a risk factor for persistent postoperative opioid use (PPOU) [5, 6, 7], beyond the expected time frame for complete recovery [8]. Persistent postoperative opioid use is now widely acknowledged as a surgical complication [9], which can be associated with harm, including physical dependence, tolerance and opioid diversion [7, 10, 11]. Therefore, opioid prescriptions for surgical pain have been recognised as a public health concern and one of the factors implicated in the opioid epidemic in the USA [12]. Accordingly, the UK's Medicines and Healthcare products Regulatory Agency has released recommendations to mitigate the risk of opioid addiction and recommended against extending opioid use for longer than 3 months in the management of acute pain [13]. Hence, it has become a significant focus for opioid‐related policy and interventions [14, 15, 16].

Minor and major surgical procedures are associated with development of PPOU [14, 17, 18, 19], but there is wide variability around its definition [9]. Studies have used time‐to‐opioid cessation [20] or presence of repeat prescriptions [21] to define PPOU. According to several studies from the USA, which defined PPOU as having one opioid prescription within the early post‐discharge period and another prescription 91–180 days after discharge, 11–17% of opioid naïve patients develop PPOU following colectomy [3, 22]. The prevalence of PPOU increases to > 30% for patients previously exposed to opioids [9, 19, 23], and this might be linked with poor surgical outcomes [6, 24] and higher healthcare costs [25].

Despite the risk of PPOU following colectomy being quantified in the USA and Canada, the external validity of these findings is limited and cannot be extrapolated to other populations due to significant variations in prescribing practices. Hence, the extent to which PPOU exists within a subset population from the UK has been hitherto unexplored. We sought to determine the prevalence of PPOU following colectomy, stratified by pre‐admission opioid exposure and identify associated PPOU predictors using linked electronic healthcare data from England. We hypothesised that the prevalence of PPOU would vary based on the individual's opioid exposure before surgery and that several predictors for PPOU could be identified.

## Methods

The study was approved by the Independent Scientific Advisory Committee and performed and reported in accordance with the strengthening the reporting of observational studies in epidemiology (STROBE) guidelines [26].

This study used linked primary and secondary care electronic databases previously described and validated [27, 28]. In brief, the Clinical Practice Research Datalink is a UK government research service that supplies anonymised electronic health records from general practices for research, and Aurum is a database containing routinely collected data, including diagnoses and prescriptions, for over 19 million people in the UK. Aurum data were linked to the Hospital Episode Statistics database which contains patient care data related to all admissions to NHS hospitals in England or care delivered in the independent sector but commissioned by the NHS. Records were coded within the dataset using a combination of International Classification of Diseases 10th revision (ICD‐10) codes for diagnosis and Office of Population Censuses and Surveys Classification of Surgical Operations and Procedures codes describing procedures performed [29]. Further linkage to Office for National Statistics data sources was required to obtain the date of death, along with linking to the 2015 Index of Multiple Deprivation [30] to get patient level deprivation index to indicate the socio‐economic status of people in this study. Index of Multiple Deprivation data were provided by Aurum from Hospital Episode Statistics and the Office for National Statistics.

**Figure 1:**
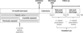
Definitions used in the study.

Patients aged ≥ 18 y were identified from Hospital Episode Statistics data by searching for procedure codes for colectomy surgeries performed between 1 January 2010 and 31 December 2019. Operations that were limited to or included the anal canal and rectum were excluded (online Supporting Information Appendix S1). Patients who did not survive the first 90 days following discharge were excluded. Eligible patients were followed up from the day of discharge to either having the study outcome of PPOU [22], end of follow up (180 days), transfer out of participating practice or date of death, whichever came first (Fig. 1, Online Supporting Information Appendix S1). To have sufficient data on pre‐operative opioid exposure, patients were excluded if they did not have a minimum of 12 months of Aurum data before the admission date for surgery.

Baseline characteristics, such as age, sex and race, were obtained from Aurum and Hospital Episode Statistics data. Race was categorised as white, black, Asian and others [31]. Comorbidities before admission were obtained from Aurum and Hospital Episode Statistics data and classified using the Charlson comorbidity index based on the number of comorbidities into 0, 1 and ≥ 2 [32]. Index of Multiple Deprivation scores [30] were categorised into quintiles from 1 to 5 (least to most deprived, respectively) [27].

A lookback window for 1 year before the date of admission was used to evaluate pre‐operative opioid exposure. Patients were considered opioid‐naïve if they did not have an opioid prescription issued in the year preceding their date of admission for surgery. They were considered ‘currently exposed’ if they were issued an opioid prescription within the 6 months before their admission date and ‘previously exposed’ if an opioid prescription was issued within 7–12 months before their date of admission, thus forming two mutually exclusive pre‐operative opioid exposed groups [33]. We categorised prescriptions as long‐acting if they included modified release or transdermal formulations or short‐acting if they had immediate release opioid formulations.

Patients were recognised as having a diagnosis of cancer if a diagnosis of colorectal cancer was reported in Hospital Episode Statistics data. Benign disease was assigned if the ICD‐10 discharge codes related to the admission included diverticular disease or inflammatory bowel disease. Patients' admissions were categorised as either emergency or elective, based on the documented indications for their surgical procedures. The surgical approaches were categorised as either open or minimally invasive, which included laparoscopic or robotic techniques using procedural codes (Y50.8, Y57.1 and Y75.2 for laparoscopic or Y75.3 for robotic, respectively).

The primary outcome was PPOU after colectomy. To identify this, early post‐discharge opioid use was defined as having at least one opioid prescription issued within 90 days of hospital discharge. This 90‐day period was selected to ensure that the opioid prescribed might be related to surgery, as the time from complete tissue healing may extend to 3 months [8]. Persistent postoperative opioid use was defined as one or more opioid prescriptions being issued within 90 days of surgical discharge along with one or more prescriptions for opioids within 91–180 days after hospital discharge (Fig. 1) [22]. This commonly used definition was chosen to allow comparison with studies from other countries that have investigated PPOU following colectomy.

Opioid prescriptions were identified using opioid product codes identified from the Aurum product dictionary, which are listed in online Supporting Information Appendix S2. Opioid codes related to sublingual buprenorphine tablets (2, 4 and 8 mg) and methadone were excluded because they are primarily prescribed as treatment for opioid addiction in the UK. Codes for injectable opioid formulations were excluded as these are typically administered by healthcare professionals and not self‐administered.

Data management and analyses were performed using STATA^®^ version 17 (StataCorp, College Station, TX, USA). Patient characteristics were stratified based on pre‐operative opioid exposure and persistent use. The proportion of patients being prescribed opioids within the early post‐discharge period and persistent users were calculated for each stratum.

Univariable and multivariable logistic regression analyses were used to examine the association of different predictors with the odds of PPOU. The analyses were stratified by pre‐operative opioid exposure as opioid‐naïve, currently exposed and previously exposed. This decision was made based on additional analysis investigating interactions between pre‐operative opioid exposure and surgical approach. The likelihood ratio test was used to check for interaction and compare coefficients between the models. Further stratification of the opioid‐naïve group by admission type was performed after detecting significant interaction between admission type and cancer‐related surgery. However, when tested on the currently and previously opioid‐exposed groups, this interaction was not significant. Age was fitted as a continuous variable; this decision was made by conducting separate models with age fitted as either a continuous or categorical variable. Then the likelihood ratio test was used to compare model fit in both models, and the variable with the best fit was selected for the final model.

We also analysed potential predictor variables, identified based on previous literature, including: sex; race; Index of Multiple Deprivation; Charlson comorbidity index; diagnosis of cancer; surgical approach; year of surgical admission; and the pre‐operative use of long‐acting opioid formulations. Unavailable Index of Multiple Deprivation values were treated as a separate category. Duration of hospital stay was not included as a predictor in the multivariable analysis because of collinearity with the surgical approach. Variables associated with the outcome in the univariable analyses (p < 0.05) were included in a multivariable manual backward logistic regression model.

## Results

**Figure 2:**
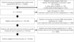
Study flow diagram. CPRD, Clinical Practice Research Datalink.

Figure 2 demonstrates the identification of the study population. Demographics of the 93,262 eligible patients who had a colectomy within the study period are shown in Table 1. Overall, the median (IQR [range]) age was 65 (51–75 [36–81]) y. There were similar proportions of men and women; 76,981 (77.0%) patients were opioid‐naïve in the year preceding their colectomy and 63,809 (68.4%) had two or more significant comorbidities. Elective admission was predominant for 66,321 (71.1%), and the most common surgical approach was open for 55,413 (59.4%) patients.

**Table 1:** Characteristics of the colectomy cohort, stratified by exposure to opioids before surgery. Values are median (IQR [range]) or number (proportion).

	Opioid naïve	Currently exposed	Previously exposed	p value*
	n = 76,981	n = 13,172	n = 3109	
Age; y	64.6 (50.2–74.5 [36.3–81.5])	68.3 (56.1–77.2 [42.5–83.2])	70.0 (57.1–78.2 [40.9–83.6])	<0.001
Sex				
Male	39,521 (51.3%)	5519 (41.9%)	1366 (44.0%)	<0.001
Female	37,460 (48.7%)	7653 (58.1%)	1743 (56.1%)	
Race				
White	70,043 (91.0%)	12,415 (94.3%)	2877 (92.5%)	<0.001
Black	1717 (2.2%)	221 (1.7%)	63 (2.0%)	
Asian	2345 (3.0%)	275 (2.0%)	100 (3.2%)	
Other	2876 (3.7%)	261 (2.0%)	69 (2.2%)	
IMD score				
1	17,739 (23.0%)	2502 (19.0%)	643 (20.7%)	<0.001
2	16,678 (21.7%)	2546 (19.3%)	671 (21.6%)	
3	15,552 (20.2%)	2672 (20.3%)	622 (20.0%)	
4	14,426 (18.7%)	2667 (20.3%)	603 (19.4%)	
5	12,479 (16.2%)	2778 (21.1%)	570 (18.3%)	
Missing	107 (0.14%)	7 (0.05%)	–	
Charlson comorbidity index				
0	20,077 (26.1%)	1954 (14.8%)	458 (14.7%)	<0.001
1	5974 (7.8%)	805 (6.1%)	185 (6.0%)	
≥2	50,930 (66.2)	10,413 (79.1%)	2466 (79.3%)	
Cancer diagnosis				
Yes	43,879 (57.0%)	6412 (48.7%)	1679 (54.0%)	<0.001
No	33,102 (43.0%)	6760 (51.3%)	1430 (46.0%)	
Admission type				
Emergency	21,802 (28.3%)	4386 (33.3%)	753 (24.2%)	<0.001
Elective	55,179 (71.7%)	8786 (66.7%)	2356 (75.8%)	
Duration of stay; days^†^				
≤3	8091 (10.5%)	873 (6.6%)	305 (9.8%)	<0.001
4–7	27,908 (36.2%)	3841 (29.2%)	1063 (34.2%)	
≥7	40,982 (53.2%)	8458 (64.2%)	1741 (56.0%)	
Surgical approach				
Open	44,674 (58.0%)	8887 (67.5%)	1852 (59.6%)	<0.001
Minimally invasive	32,307 (41.9%)	4285 (32.5%)	1257 (40.4%)	
Year of surgery				
2010	7017 (8.4%)	1392 (10.6%)	276 (8.9%)	<0.001
2011	7208 (8.6%)	1355 (10.3%)	333 (10.7%)	
2012	7566 (9.1%)	1396 (10.6%)	309 (9.9%)	
2013	7718 (9.2%)	1342 (10.2%)	317 (10.2%)	
2014	7826 (9.4%)	1326 (10.1%)	312 (10.0%)	
2015	8346 (10.0%)	1343 (10.2%)	331 (10.7%)	
2016	8745 (10.5%)	1325 (10.1%)	327 (10.5%)	
2017	8980 (10.8%)	1341 (10.2%)	314 (10.1%)	
2018	9708 (11.6%)	1261 (9.6%)	308 (9.9%)	
2019	10,341 (12.4%)	1091 (8.3%)	282 (9.1%)	
Practice region				
North‐east	2640 (3.4%)	743 (5.6%)	152 (4.9%)	<0.001
North‐west	12,061 (15.7%)	2624 (19.9%)	560 (18.1%)	
Yorkshire and Humber	2843 (3.7%)	559 (4.2%)	121 (3.9%)	
East Midlands	1822 (2.4%)	313 (2.4%)	79 (2.5%)	
West Midlands	13,093 (17.0%)	2609 (19.8%)	593 (19.1%)	
East of England	3621 (4.7%)	516 (3.9%)	128 (4.1%)	
South‐west	10,616 (13.8%)	1832 (13.9%)	420 (13.5%)	
South central	10,014 (13.1%)	1550 (11.8%)	379 (12.2%)	
London	12,878 (16.7%)	1325 (10.1%)	404 (12.9%)	
South‐east coast	7375 (9.6%)	1101 (8.3%)	273 (8.8%)	
Long‐acting opioid				
Yes	–	1972 (15.0%)	152 (4.9%)	<0.001
No	–	11,200 (85.0%)	2956 (95.1%)	

IMD, Index of Multiple Deprivation.

* All p values were obtained using the chi‐square test except for the median age for which the Kruskal–Wallis test was used.

† Duration of hospital stay calculated as the number of days from the first day of admission to the day of discharge.

At least one opioid prescription was issued to 15,081 (16.2%) patients within 90 days of surgical discharge. Of these, 6791 (45.0%) patients were opioid‐naïve, 7528 (49.9%) were currently exposed and 762 (5.0%) were previously exposed. Among each category of pre‐operative opioid exposure in the overall colectomy cohort, 6791 (8.8%) of opioid‐naïve, 7528 (57.2%) of those currently exposed and 762 (24.5%) of previously exposed received opioid prescriptions after discharge (Table 2).

**Table 2:** Patients having early and persistent opioid use post‐discharge. Values are number (proportion).

		Opioid naïve	Currently exposed	Previously exposed	p value*
		n = 76,981	n = 13,172	n = 3109	
Early post discharge opioid use	Yes	6791 (8.8%)	7528 (57.2%)	762 (24.5%)	<0.001
	No	70,190 (91.2%)	5644 (42.8%)	2347 (75.5%)	
Persistent opioid use	Yes	1918 (2.5%)	5317 (40.4%)	305 (9.8%)	<0.001
	No	75,063 (97.5%)	7855 (59.6%)	2804 (90.2%)	

* Chi‐square test.

In this cohort of patients who underwent colectomy, 7540 (8.1%) developed PPOU. Patients with pre‐operative opioid exposure had the highest persistent use (p < 0.001): 5317 (40.4%) in the currently exposed group; 305 (9.8%) in the previously exposed group; and 1918 (2.5%) in the opioid‐naïve group (Table 2).

For patients in the opioid‐naïve group for both admission types, predictors associated with higher odds of PPOU included living in the most deprived quintile and having a high Charlson comorbidity index. Conversely, minimally invasive surgery was associated with significantly lower odds of PPOU in opioid‐naïve patients for both emergency and elective admissions. Variation over time was also present, with significantly lower odds of having opioid prescriptions from 2016 to 2019. Female sex and cancer surgery (adjusted odd ratio (aOR) 1.24, 95%CI 1.05–1.46) were only linked to higher odds of PPOU in the emergency setting (Table 3).

**Table 3:** Univariable and multivariable logistic regression analysis investigating the predictors of persistent post‐discharge opioid use for opioid naïve patients (n = 76,981), by surgical admission type. Values are OR (95%CI).

Predictors	Emergency			Elective		
	n = 21,802			n = 55,179		
	Univariable analysis	Multivariable analysis		Univariable analysis	Multivariable analysis	
	OR (95%CI)	OR (95%CI)	p value*	OR (95%CI)	OR (95%CI)	p value*
Age; y	1.01 (1.01–1.02)	1.00 (0.99–1.00)	0.192	1.01 (1.00–1.01)	0.99 (0.99–1.00)	0.595
Sex						
Male	reference	reference	–	reference	reference	–
Female	1.05 (0.90–1.21)	1.04 (0.89–1.21)	0.589	0.78 (0.69–0.88)	0.85 (0.76–0.96)	0.010
Race						
White	reference	reference	–	reference	reference	–
Black	0.56 (0.31–1.03)	0.54 (0.29–0.99)	0.050	1.16 (0.80–1.69)	1.11 (0.76–1.63)	0.564
Asian	0.79 (0.50–1.26)	0.84 (0.53–1.35)	0.486	1.09 (0.79–1.51)	1.18 (0.85–1.63)	0.320
Other	0.58 (0.36–0.94)	0.67 (0.41–1.09)	0.113	0.91 (0.66–1.25)	1.08 (0.78–1.48)	0.641
IMD						
1 (least deprived)	reference	reference	–	reference	reference	–
2	1.18 (0.92–1.51)	1.20 (0.93–1.54)	0.142	1.02 (0.85–1.22)	1.03 (0.85–1.22)	0.780
3	1.27 (0.99–1.63)	1.29 (1.12–1.66)	0.037	1.17 (0.98–1.40)	1.16 (0.97–1.39)	0.087
4	1.48 (1.16–1.88)	1.60 (1.26–2.04)	0.001	1.16 (0.96–1.39)	1.16 (0.97–1.40)	0.098
5 (most deprived)	1.37 (1.07–1.75)	1.45 (1.13–1.86)	0.003	1.46 (1.21–1.75)	1.45 (1.21–1.74)	0.001
Charlson comorbidity index						
0	reference	reference	–	reference	reference	–
1	1.64 (1.25–2.15)	1.55 (1.17–2.04)	0.002	1.96 (1.45–2.64)	1.88 (1.38–2.56)	0.001
≥2	1.96 (1.65–2.31)	1.83 (1.53–2.18)	0.001	2.61 (2.13–3.20)	2.45 (1.96–3.06)	0.001
Cancer diagnosis						
No	reference	reference	–	reference	reference	–
Yes	0.03 (0.02–0.03)	1.24 (1.05–1.46)	0.008	1.44 (1.26–1.64)	1.11 (0.95–1.29)	0.172
Surgical approach						
Open	reference	reference	–	reference	reference	–
Minimally invasive	0.58 (0.44–0.76)	0.66 (0.51–0.87)	0.003	0.62 (0.55–0.70)	0.699 (0.62–0.78)	0.001
Year of surgery						
2010	reference	reference	–	reference	reference	–
2011	0.92 (0.67–1.27)	0.89 (0.65–1.23)	0.499	1.13 (0.89–1.42)	1.12 (0.89–1.41)	0.326
2012	1.01 (0.74–1.38)	0.99 (0.73–1.35)	0.955	0.86 (0.68–1.10)	0.87 (0.69–1.12)	0.296
2013	0.81 (0.59–1.11)	0.81 (0.89–1.11)	0.190	0.84 (0.65–1.07)	0.87 (0.68–1.11)	0.268
2014	0.75 (0.53–1.04)	0.74 (0.53–1.03)	0.073	0.72 (0.56–0.92)	0.74 (0.58–0.95)	0.021
2015	0.73 (0.53–1.01)	0.74 (0.54–1.03)	0.072	0.69 (0.54–0.88)	0.73 (0.57–0.94)	0.017
2016	0.64 (0.46–0.88)	0.64 (0.46–0.88)	0.008	0.54 (0.42–0.70)	0.57 (0.44–0.75)	0.001
2017	0.62 (0.45–0.87)	0.62 (0.44–0.86)	0.005	0.47 (0.36–0.62)	0.51 (0.39–0.67)	0.001
2018	0.52 (0.37–0.72)	0.52 (0.37–0.73)	0.001	0.48 (0.37–0.63)	0.52 (0.40–0.68)	0.001
2019	0.53 (0.38–0.75)	0.55 (0.38–0.77)	0.001	0.44 (0.34–0.57)	0.48 (0.36–0.63)	0.001

IMD, Index of Multiple Deprivation.

* p values obtained from multivariable analysis.

For the currently exposed group, pre‐operative use of long‐acting opioid formulations was associated with significantly greater odds of PPOU than taking short‐acting opioids (aOR 3.41, 95%CI 3.07–3.77). Female patients had higher odds of developing PPOU (aOR 1.13, 95%CI 1.05–1.22). Other predictors associated with higher odds included high deprivation index and high Charlson comorbidity index (Table 4). Conversely, black, Asian and other races had lower odds of developing PPOU than white race. In contrast with the opioid‐naïve group, a diagnosis of cancer was associated with lower odds of PPOU (aOR 0.84, 95%CI 0.77–0.91).

**Table 4:** Univariable and multivariable logistic regression analysis investigating the predictors of persistent post‐discharge opioid use for previously exposed patients (n = 16,281) in the post‐discharge period following colectomy. Values are OR (95%CI).

Predictors	Currently exposed			Previously exposed		
	n = 13,172			n = 3109		
	Univariable analysis	Multivariable analysis		Univariable analysis	Multivariable analysis	
	OR (95%CI)	OR (95%CI)	p value*	OR (95%CI)	OR (95%CI)	p value*
Age; y	1.00 (1.00–1.01)	1.00 (1.00–1.01)	0.002	0.99 (0.98–1.00)	0.99 (0.98–0.99)	0.019
Sex						
Male	reference	reference	–	reference	reference	–
Female	1.19 (1.11–1.28)	1.13 (1.05–1.22)	<0.001	1.27 (0.99–1.61)	1.23 (0.96–1.57)	0.104
Race						
White	reference	reference	–	reference	reference	–
Black	0.71 (0.53–0.95)	0.64 (0.48–0.85)	0.003	0.95 (0.41–2.22)	0.80 (0.34–1.91)	0.632
Asian	0.72 (0.55–0.92)	0.72 (0.55–0.93)	0.015	0.68 (0.31–1.48)	0.56 (0.25–1.23)	0.152
Other	0.63 (0.49–0.83)	0.69 (0.53–0.91)	0.010	0.71 (0.28–1.77)	0.65 (0.25–1.65)	0.370
IMD						
1 (least deprived)	reference	reference	–	reference	reference	–
2	1.08 (0.96–1.21)	1.08 (0.97–1.22)	0.173	1.16 (0.79–1.70)	1.15 (0.78–1.70)	0.477
3	1.22 (1.10–1.36)	1.23 (1.09–1.37)	<0.001	1.17 (0.78–1.73)	1.08 (0.73–1.61)	0.677
4	1.32 (1.18–1.48)	1.36 (1.21–1.52)	<0.001	1.42 (0.97–2.08)	1.38 (0.93–2.03)	0.100
5 (most deprived)	1.67 (1.50–1.87)	1.73 (1.54–1.93)	<0.001	1.49 (1.02–2.18)	1.41 (0.95–2.08)	0.083
Missing	0.74 (0.14–3.81)	0.73 (0.14–3.87)	0.716	–	–	–
Charlson comorbidity index						
0	reference	reference	–	reference	reference	–
1	1.24 (1.05–1.48)	1.23 (1.03–1.47)	0.024	1.33 (0.76–2.33)	1.71 (0.95–3.05)	0.069
≥2	1.61 (1.45–1.78)	1.46 (1.30–1.64)	<0.001	1.14 (0.81–1.63)	1.52 (1.03–2.25)	0.035
Cancer diagnosis						
No	reference	reference	–	reference	reference	–
Yes	0.93 (0.87–1.00)	0.84 (0.77–0.91)	<0.001	0.77 (0.61–0.98)	0.89 (0.68–1.19)	0.454
Surgical approach						
Open	reference	reference		reference	reference	
Minimally invasive	0.96 (0.89–1.03)	0.99 (0.92–1.08)	0.940	0.66 (0.51–0.85)	0.72 (0.54–0.94)	0.017
Admission type						
Emergency	reference	reference	–	reference	reference	–
Elective	1.14 (1.06–1.23)	1.22 (1.13–1.33)	<0.001	0.71 (0.55–0.93)	0.79 (0.59–1.06)	0.124
Year of surgery						
2010	reference	reference	–	reference	reference	–
2011	0.96 (0.82–1.12)	0.95 (0.82–1.11)	0.537	0.72 (0.44–1.15)	0.71 (0.43–1.15)	0.166
2012	0.87 (0.75–1.02)	0.85 (0.73–0.99)	0.047	0.64 (0.38–1.05)	0.63 (0.38–1.03)	0.070
2013	1.08 (0.93–1.26)	1.07 (0.91–1.27)	0.376	0.68 (0.42–1.12)	0.69 (0.43–1.13)	0.148
2014	0.93 (0.79–1.08)	0.88 (0.76–1.07)	0.146	0.41 (0.24–0.72)	0.42 (0.24–0.73)	0.002
2015	0.99 (0.85–1.16)	0.95 (0.81–1.12)	0.597	0.59 (0.36–0.97)	0.57 (0.35–0.95)	0.031
2016	0.95 (0.81–1.11)	0.93 (0.79–1.09)	0.383	0.60 (0.36–0.98)	0.59 (0.36–0.97)	0.040
2017	0.97 (0.83–1.23)	0.92 (0.79–1.08)	0.356	0.41 (0.24–0.72)	0.41 (0.23–0.72)	0.002
2018	1.00 (0.96–1.17)	0.97 (0.83–1.14)	0.724	0.55 (0.33–0.92)	0.55 (0.33–0.94)	0.029
2019	1.08 (0.93–1.28)	1.05 (0.88–1.23)	0.596	0.71 (0.43–1.17)	0.73 (0.43–1.21)	0.223
Long‐acting opioid						
No	reference	reference	–	reference	reference	–
Yes	3.50 (3.16–3.87)	3.41 (3.07–3.77)	<0.001	0.85 (0.47–1.52)	0.86 (0.47–1.54)	0.608

IMD, Index of Multiple Deprivation.

* p values obtained from multivariable analysis.

In the previously exposed group, having two or more comorbidities was the only predictor associated with higher odds of PPOU. Compared with open colectomy, minimally invasive surgery was associated with lower odds of PPOU (aOR 0.72, 95%CI 0.54–0.94). Patients who had a colectomy performed between 2014 and 2018 also had lower odds for PPOU compared with colectomy performed in 2010. The use of long‐acting opioid formulations before colectomy was not associated with developing PPOU in this cohort (Table 4).

## Discussion

This nationwide study in patients undergoing colectomy in England contributes to a growing body of literature on post‐discharge opioid use after surgery [34, 35]. Our stratified analysis based on pre‐operative opioid exposure has enabled quantification of the risk of PPOU with identification of predictors for developing this complication in three different groups of patients.

Our findings show that 16.2% of patients were issued prescriptions for opioid analgesics within 90 days of discharge. This finding aligns with a study from the USA of 367 patients that reported a similar proportion (15%) of patients having post‐discharge opioids following colorectal surgery [36]. However, it contrasts with the results from other population‐based studies that examined opioid use after various surgical procedures. Another USA‐based study found that 80.3% of patients received post‐discharge opioid prescriptions after a broad range of surgical procedures, including colectomy [37, 38]. Additionally, a study by Ladha et al. reported that the rate of filled opioid prescriptions following low‐risk abdominal surgical procedures was seven times higher in the USA and Canada than in Sweden, where only 11% of patients were given post‐discharge opioids [38], which is more consistent with our findings.

Half of the patients in our cohort who were discharged from the hospital with a prescription for opioids (8.1% of the overall cohort) and continued to be prescribed opioids for up to 180 days following discharge. This overall finding was lower than the 10% prevalence reported in a prospective study from the USA [3] and the figures determined by a USA database analysis showing PPOU rates ranging between 13.5% and 21.2% following colectomy [39]. Furthermore, among opioid‐naïve patients, 2.5% developed PPOU. This finding aligns with that reported by Clarke et al. [17], who used the same definition of PPOU in a study that included different types of abdominopelvic procedures and was not strictly limited to colectomy.

We found that patients with pre‐operative opioid exposure accounted for the majority of persistent users. This result is similar to that of previous studies showing that PPOU is more common in patients with a history of opioid exposure before surgery [3, 24, 40], although pre‐operative opioid exposure is not defined consistently in terms of dose, recency, duration and continuity of use. While the definition adopted in the present study did not require evidence of long‐term opioid use before surgery, a large proportion of patients in this group continued to use opioids for more than 90 days following discharge.

The odds of persistent opioid use were more than three times higher among individuals who used long‐acting opioid formulations in the 180 days before colectomy than those who used short‐acting formulations [6, 20, 41]. This finding contributes to the growing body of evidence suggesting that long‐acting and modified‐release formulations are a modifiable risk factor for PPOU [42].

The association between PPOU and pre‐operative opioid exposure is likely to be multifactorial. One possible explanation is that patients with previous opioid exposure can develop tolerance or hyperalgesia, which may make the management of their postoperative pain more challenging and lead to persistent use [43, 44]. Another possible explanation is that patients who were taking opioids pre‐operatively had already adjusted to opioid‐related adverse effects such as nausea, vomiting and constipation, while these may have discouraged their opioid‐naïve counterparts from continuing their opioids. In addition to the currently exposed group, we also included patients with previous opioid exposure. This is a distinct group with a potentially different trajectory of PPOU that is often overlooked. We found that despite their remote exposure to opioids before surgery, these patients were still at greater risk of PPOU than those in the naïve group. Although we did not find an association between the use of long‐acting opioid formulations and PPOU in this group of patients, it is essential to note that this finding may be limited by the small sample size in this group.

This extensive electronic health records analysis also reveals that among opioid‐naïve and previously exposed patients with a history of remote opioid exposure, a minimally invasive surgical approach was associated with significantly lower odds of PPOU than an open approach. In contrast, this protective effect was not seen in the currently exposed group. This finding supports literature from the USA demonstrating that minimally invasive techniques attenuate the odds of developing PPOU and should be considered when skills and resources are available, especially for opioid‐naïve patients [3, 39, 45]. A possible explanation for this association may be that a minimally invasive approach is associated with reduced incision length [45] and less inflammation and nerve damage [46], which may lead to lower levels of incisional pain and analgesic requirements. However, other studies examining the effect of surgical approach on opioid consumption have yielded contradictory findings that do not fully support this theory. For instance, while single‐institution studies show decreased inpatient opioid use after minimally invasive vs. open surgery [47, 48], Vu et al. [49] reported no difference in post‐discharge opioid consumption by patients undergoing colectomies performed by these two approaches across many institutions. Additional factors may confound this finding including variations in surgical technique and enhanced recovery protocols, especially given inconsistent reductions in PPOU associated with minimally invasive surgery [50]. Further prospective studies are needed to assess the possible benefits of minimally invasive approaches on PPOU in specific surgical populations and pre‐operative opioid use groups.

Having two or more comorbidities increased the odds of PPOU among all groups, while those in the most deprived quintiles had increased odds of PPOU in opioid‐naïve patients and current users. These results align with previous studies that have evaluated these factors in major abdominal surgical procedures [17, 22].

Variation was observed between racial groups. Opioid‐naïve patients of black race had significantly lower odds of developing PPOU, when compared with patients of white race, while current opioid users of white race were at higher risk of becoming persistent users compared with all other races. Previous research has identified racial disparities in pain diagnosis and treatment [51], and white patients are more likely to be prescribed opioids than black patients [52]. In light of this evidence, we have to consider that the present study's findings may have been confounded by clinicians' implicit bias in the assessment of pain severity and choice of treatment, implicit bias related to repeat opioid prescriptions [53], hospital‐level factors and surgical setting.

Over the 10‐year study period, there were several changes to clinical practice that may have impacted the prescription of opioids and the incidence of PPOU. These changes include: widespread implementation of enhanced recovery programmes [54]; increased use of multimodal and opioid‐sparing analgesia [55]; regional and neuraxial anaesthesia; and increased uptake of minimally invasive surgery. Additionally, there has been an increased awareness of the potential problems associated with opioids, which may have led to more responsible prescribing and stewardship practices.

Our study has several limitations. First, although Aurum has longitudinal data on opioid prescription records before and after surgery, limiting the possibility of recall bias, it lacks clinical details such as in‐hospital drug therapy, patient‐reported outcome measures, and some complications (such as persistent postsurgical pain). Moreover, the assessment of PPOU using electronic health record data is limited by the inability to measure whether drugs in prescriptions dispensed were subsequently taken by the patients. Nevertheless, despite these limitations, the use of prescription data as a proxy for confirmed drug consumption is widespread in drug utilisation research [19].

Additionally, data obtained from Hospital Episode Statistics lack information on hospital‐level factors such as pre‐operative preparation, use of regional anaesthesia and availability of enhanced recovery protocols. It is unknown whether opioid prescribing guidance and discharge opioid tapering instructions were available for patients. While Daliya et al. [35] previously acknowledged the lack of these resources within hospitals in England, implementing these services along with opioid stewardship programmes may be effective for minimising post‐discharge opioid prescribing [56, 57].

Another limitation is the lack of information on drugs prescribed privately or obtained via other sources. In addition, during the study period, some ‘weak’ opioids, such as dihydrocodeine and codeine, were available without a prescription, which may have led to the under‐representation of the prevalence of PPOU related to these opioids. We did not study all the risk factors for PPOU reported in the literature such as history of depression, anxiety and pre‐operative benzodiazepine and antidepressant use [6, 7]. The dose, duration and type of opioids used before surgery may also be associated with the development of PPOU [7], but these factors were not tested in the current analysis. Additionally, we could not control for factors affecting the choice of surgical approach or admission type. However, we identified several patient‐ and surgery‐specific predictors associated with long‐term opioid use that had not been identified previously in a population from the UK.

After undergoing colectomy in hospitals across England, 8.1% of patients continued to receive opioid prescriptions more than 3 months after discharge. Persistent postoperative opioid use was more common in patients with pre‐operative opioid exposure. Importantly, a minimally invasive surgical approach was associated with lower odds of PPOU in opioid naïve and previously exposed patients compared with open colectomy and may present a modifiable risk factor meriting more clinical attention.

## Supporting information


**Appendix S1.** OPCS and ICD codes used to identify colectomy, inflammatory bowel disease and diverticular disease.

**Appendix S2.** Opioid products codes.
