# Supplementary material for: Predictors of persistent postoperative opioid use following colectomy: a population‐based cohort study from England
Source: Anaesthesia. 2023 Jun 2;78(9):1081–92. doi: 10.1111/anae.16055 (PMC10953341; doi:10.1111/anae.16055)
Supplement: Supplementary file 1 — Appendix S1. OPCS and ICD codes used to identify colectomy, inflammatory bowel disease and diverticular disease. [file ANAE-78-1081-s002.docx]

**Appendix S1 –** OPCS and ICD codes used to identify colectomy, inflammatory bowel disease and diverticular disease

**Colectomy codes**

H041, H042, H043, H048, H049, H051, H052, H053, H058, H059, H061, H062, H063, H064, H068, H069, H071, H072, H073, H074, H078, H079, H081, H082, H083, H084, H085, H088, H089, H091, H092, H093, H094, H095, H098, H099, H101, H102, H103, H104, H105, H108, H109, H111, H112, H113, H114, H115, H118, H119, H291, H292, H293, H294, H298, H299, H331, H332, H333, H334, H335, H336, H337, H338, H339

**Inflammatory bowel disease codes**

K50, K500, K501, K508, K509, K51, K510, K512, K513, K514, K515, K518, K519, K520, K521, K522, K523, K528, K529

**Diverticular disease codes**

K57,K570, K571, K572, K573, K574, K575, K578, K579
